# Potency of propofol for inducing loss of consciousness in end-stage kidney disease patients

**DOI:** 10.1371/journal.pone.0254520

**Published:** 2021-08-12

**Authors:** Mi Roung Jun, Mun Gyu Kim, Ki Seob Han, Ji Eun Park, Ho Bum Cho, Sun Young Park, Sanghoon Song, Jae Hwa Yoo, Ji Won Chung, Sang Ho Kim

**Affiliations:** 1 Department of Anesthesiology and Pain Medicine, Samsung Changwon Hospital, Sungkyunkwan University School of Medicine, Changwon, Republic of Korea; 2 Department of Anaesthesiology and Pain Medicine, Soonchunhyang University Hospital Seoul, Seoul, Republic of Korea; Universidade Estadual Paulista Julio de Mesquita Filho, BRAZIL

## Abstract

It can be difficult for anesthesiologists to determine the optimal dose of propofol for end-stage kidney disease (ESKD) patients due to changes in drug disposition. The purpose of this study was to evaluate the potency of propofol for inducing loss of consciousness in ESKD patients. Patients with normal kidney function (Control group, n = 15), those with ESKD (ESKD group, n = 15), and those with ESKD undergoing cervical epidural anesthesia (ESKD-CEB group, n = 15) were administered propofol by target-controlled infusion (TCI) using the Schneider model. The effect-site concentration (*Ce*) of propofol started at 0.5 μg/ml and increased in increments of 0.5 μg/ml until the patient did not respond to verbal commands. The relationship between the probability (*P*) of loss of consciousness and the *Ce* of propofol was analyzed in each group using logistic regression. The *Ce* values of propofol at the time of loss of consciousness were 4.3 ± 0.9, 3.7 ± 0.9, and 3.3 ± 1.0 *μ*g/ml for the Control, ESKD, and ESKD-CEB* groups, respectively (*significant difference vs. control, *P* < 0.05). The estimated *Ce*_*50*_ values for lost ability to respond to verbal command were 4.56, 3.75, and 3.21 *μ*g/ml for the Control, ESKD, and ESKD-CEB groups, respectively. In conclusion, when inducing anesthesia in ESKD patients, we recommend using an initial dose similar to that of patients with normal kidney function, or rather starting with a lower dose.

## Introduction

Propofol is a short-acting, lipophilic intravenous general anesthetic. The hypnotic action of propofol is probably mediated through γ-aminobutyric acid (GABA) receptor (agonist) and N-methyl-D-aspartate (NMDA) receptor (antagonist). Propofol has a protein binding of about 98% and is rapidly metabolized to water-soluble inactive metabolites in the liver and excreted through the kidneys [1, 2].

End-stage kidney disease (ESKD) is defined as irreversible decline in a person’s own kidney function, which is severe enough to be fatal in the absence of dialysis or transplantation [3]. Abnormal increases in toxins, inflammatory factors, and parathyroid hormone (PTH) can lead to uremic symptoms in patients with ESKD, which can in turn affect drug disposition [4, 5]. For example, the volume of distribution (Vd) may increase due to interference with protein binding [4]. Thus, an appropriate dose of a drug in patients with normal kidney function can appear inadequate or excessive in ESKD patients.

Propofol is commonly used for sedation and general anesthesia in ESKD patients due to rapid recovery after continuous infusion. Previous studies have shown that the pharmacokinetics of propofol are not significantly different between patients with normal kidney function and ESKD [6–8]. Although some investigators have suggested that the hyperdynamic circulation caused by anemia increases the dose requirement of propofol for inducing anesthesia in ESKD patients [9], studies are conflicting and controversial.

It is important to determine the optimal initial dose of propofol, as this drug can cause cardiovascular instability during the induction of anesthesia. However, information about the concentration-response relationship of propofol during induction of anesthesia in ESKD patients is limited.

This study was performed to investigate the potency of propofol for loss of consciousness, administered by target-controlled infusion (TCI) for inducing general anesthesia in ESKD patients compared to patients with normal kidney function. In addition, we evaluated the potency of propofol for inducing loss of consciousness in ESKD patients who received a cervical epidural block (CEB).

## Materials and methods

### Patient population

This study was approved by the Institutional Review Board of hospital (Ref. 2016-05-006-003), and was registered with the International Clinical Trials Registry Platform (http://cris.nih.go.kr). Written informed consent was obtained from all patients on the day before surgery. Fifteen patients with normal kidney function scheduled for elective surgery under general anesthesia were recruited as the control group (Control group). Thirty patients with ESKD presenting for arteriovenous fistulae surgery were enrolled between October 2016 and April 2018. Fifteen of the thirty patients with ESKD received general anesthesia (ESKD group); and the other fifteen patients received a CEB and underwent monitored anesthesia care (ESKD-CEB group). All ESKD patients had been dialyzed the day before surgery. Preoperative laboratory testing was performed before surgery in all patients, and after dialysis in the ESKD patients. Patients were excluded for the following reasons: a history of neurological or psychological disease or the presence of current neurological symptoms; the presence of a liver function abnormality; body mass index ≥ 30 kg/m^2^; a history of hearing impairment; a history of an allergic reaction to soybean; a history of an adverse drug reaction to propofol or local anesthetic; or kidney transplantation. Patients who were on medications that affected the central nervous system (CNS) (e.g., analgesics, anticonvulsants, and hypnotics) were also excluded from the study.

### Study procedure

Electrocardiography, noninvasive arterial blood pressure, and peripheral oxygen saturation monitoring were initiated when patients arrived in the operating room. An epidural catheter was inserted at C7-T1, and 15 ml of 0.45% ropivacaine was administered after negative aspiration in the ESKD-CEB group. Adequate sensory block was confirmed 20 min after injecting the local anesthetic; CNS symptoms (e.g., dizziness, visual and auditory disturbances, disorientation, drowsiness, tremors, shivering, muscular twitching, and generalized tonic-clonic convulsions) caused by the local anesthetics were observed and recorded.

In all patients, disposable bispectral index (BIS) sensors were placed on the forehead, as recommended by the manufacturer, and connected to a BIS monitor (BIS^®^ Monitor; Medtronic, Dublin, Ireland). The effect-site concentration (*Ce*) of propofol started at 0.5 *μ*g/ml and was delivered via a TCI pump (Orchestra^®^ Base Primea; Fresenius Vial, Brézins, France) using the Schnider model [10]. The *Ce* was increased in increments of 0.5 *μ*g/ml, 3 min after the previous target *Ce* was reached, until the patient did not respond to the loud verbal command, “open your eyes”, which was defined as loss of consciousness (LOC). The BIS values, blood pressure, heart rate, peripheral oxygen saturation, and total amount of propofol infused were recorded 3 min after the target *Ce* was reached, and the investigator then evaluated the level of sedation. Oxygen was administered at 5 L/min via a face mask during the study period. Adverse events requiring cardiovascular or respiratory support during propofol-induced sedation were observed and recorded.

### Sample size

In the preliminary study, the total amount of propofol at the time of loss of consciousness were 156.4 ± 15.0 mg, 82.6 ± 8.6 mg, and 63.9 ± 15.0 mg for the Control, ESKD, and ESKD-CEB groups, respectively. Based on this, the sample sizes required to confirm the difference in the total amount of propofol between the Control and ESKD groups, ESKD and ESKD-CEB groups, and Control and ESKD-CEB groups was 11 (α = 0.05, β = 0.2). A 30% dropout rate was applied here, and the final sample size was 15 subjects per group. The power of the sample size was reconfirmed using R package wmwpow.

### Statistics

One-way analysis of variance or the Kruskal-Wallis test, followed by multiple comparison procedures using the Holm-Sidak method, was performed to compare the three groups. Continuous variables are presented as mean ± standard deviation, and categorical variables as integers. All statistical analyses were performed using SigmaPlot 13.0 for Windows (Systat Software, Inc., Chicago, IL, USA). A P-value < 0.05 was considered significant. We used R package wmwpow for power analysis.

### Probability of LOC and pharmacodynamic analysis

The following sigmoid E_max_ model was used to determine the relationship between the probability (*P*) of LOC and the *Ce* of propofol in each group:

$$p\;of\;LOC=\frac{C_{e}^{\gamma }}{C_{e_{50\_Group}}^{\gamma }+C_{e}^{\gamma }}$$

Where Ce_50_Group_ is the Ce associated with a 50% probability of LOC in each study group, and γ is the slope of the concentration versus the unconscious probability curve. The pharmacodynamic model parameters were estimated using the “LIKELIHOOD LAPLACE METHOD = conditional” option in NONMEM® 7 level 3 (ICON Development Solutions, Dublin, Ireland). The IIV(random inter-individual effect) of the Ce_50_ was fixed at zero.

## Results

**Fig 1:**
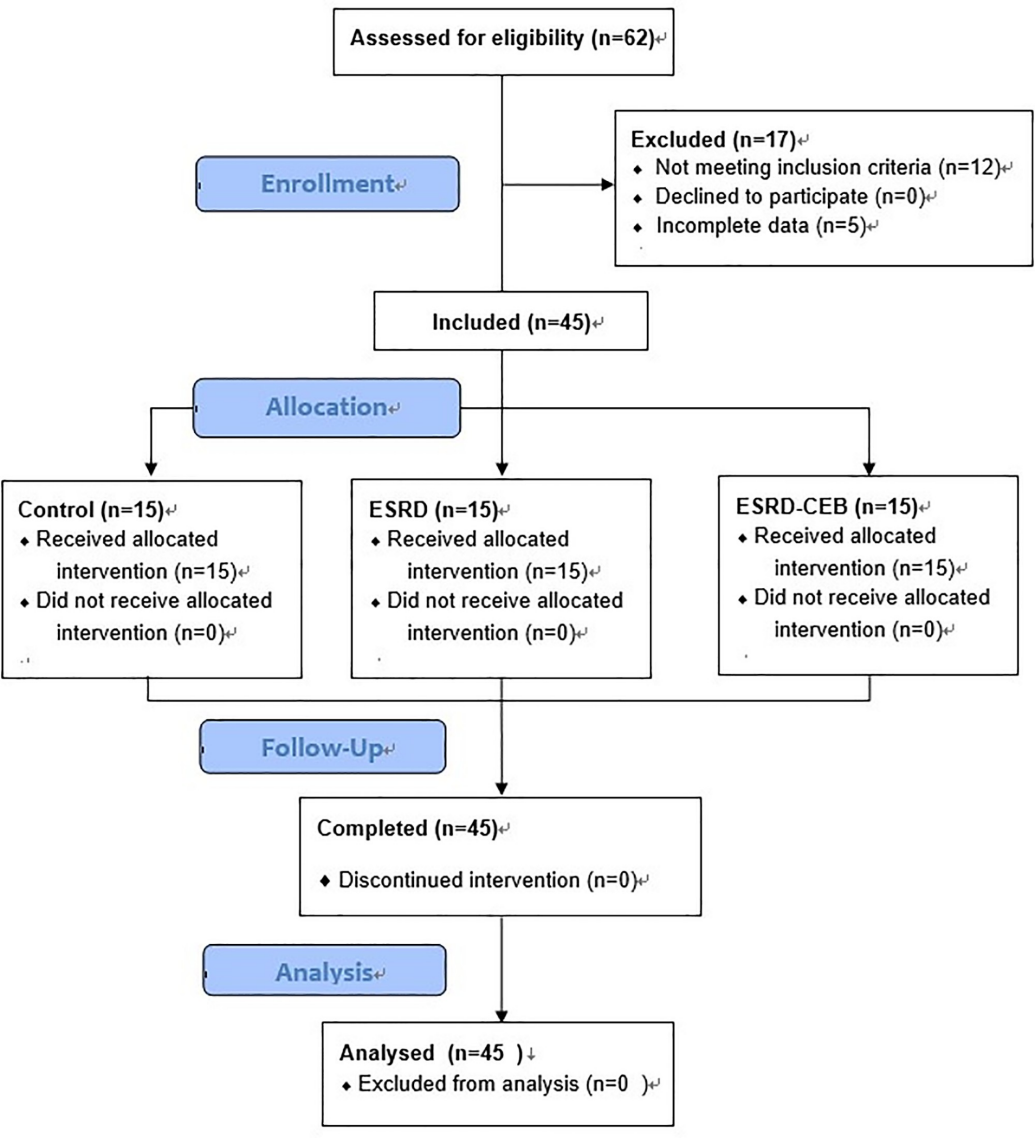
The flow diagram of the study.

The study flow diagram is shown in Fig 1. No signs of CNS toxicity due to the local anesthetic were detected during the CEB in any patients in the ESKD-CEB group. No significant adverse events requiring cardiovascular or respiratory support occurred during propofol-induced sedation. The patients’ characteristics are summarized in Table 1. There were expected differences in laboratory measures of renal function, hemoglobin, and platelets. In addition, in the ESKD patients who had received a CEB were older with lower BMI and weight. Neither lean body mass or albumin was different.

**Table 1:** Patient characteristics and preoperative laboratory values.

	Control	ESKD	ESKD_CEB	*P*
Age (yr)	49.7 ± 9.5	59.1 ± 13.9	66.1 ± 11.6*	0.002
Sex, M/F	8/7	6/9	7/8	
Height (cm)	165.7 ± 9.0	157.4 ± 7.1*	159.0 ± 8.1	0.023
Weight (kg)	68.4 ± 12.6	56.4 ± 9.9*	52.4 ± 11.0*	0.002
Body mass index (kg/m^2^)	24.8 ± 3.3	22.8 ± 3.7	20.5 ± 3.9*	0.016
Lean body mass (kg)	49.1 (34.3–49.3)	43.0 (37.8–46.0)	43.4 (37.9–57.5)	0.132
Albumin (g/dL)	4.6 (4.2–4.6)	4.3 (4.0–4.4)	4.4 (4.1–4.8)	0.184
Hemoglobin (g/dL)	13.9 ± 1.4	10.7 ± 2.0*	11.9 ± 1.4*†	<0.001
Platelet (ⅹ10^3^/μL)	247 ± 82	162 ± 71*	177 ± 45*	0.003
BUN (mg/dL)	9.7 (36.2–74.7)	48.9 (36.5–62.7)*	54.1 (8.0–14.2)*	<0.001
Creatinine (mg/dL)	0.8 (0.7–0.8)	8.8 (5.1–9.8)*	7.4 (4.9–11.1)*	<0.001
eGFR (ml/min/1.73m^2^)	101.9 (97.6–111.2)	5.3 (4.4–9.2)*	7.2 (3.7–9.9)*	<0.001
Potassium (mmol/L)	4.3 ± 0.7	4.9 ± 0.5*	4.8 ± 0.9	0.031
Prothrombin time (INR)	1.1 (1.0–1.1)	1.0 (0.9–1.1)*	1.0 (1.0–1.0)*	0.015

Values expressed in mean ± SD, median (25th–75th percentiles) or numbers of patients. ESKD, End stage kidney disease; CEB, Cervical epidural block; eGFR, Estimated glomerular filtration rate; INR, International normalized ratio.

*There is significant difference vs Control (*P* < 0.05)

†There is significant difference vs ESKD (*P* < 0.05).

**Fig 2:**
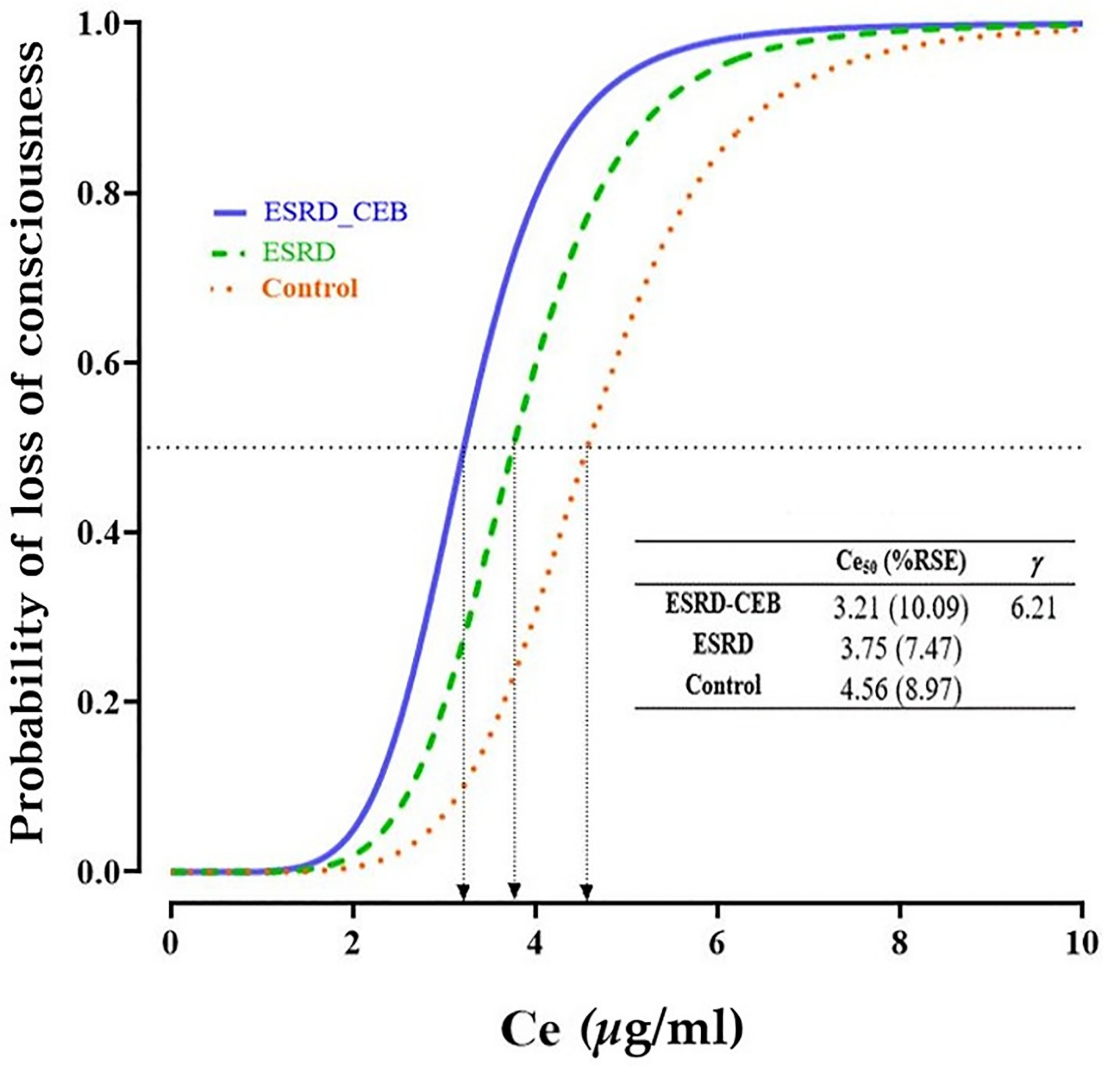
Relationship between the probability of LOC and the Ce of propofol at each study groups. Ce_50_, effect-site concentration associated with a 50% probability of LOC; ESRD, end stage renal disease; LOC, loss of consciousness; CEB, cervical epidural block; γ, steepness of the concentration versus response relationship; RSE, relative standard error.

The *Ce* values of propofol at the time of loss of consciousness were 4.3 ± 0.9, 3.7 ± 0.9, and 3.3 ± 1.0 *μ*g/ml for the Control, ESKD, and ESKD-CEB groups, respectively. The *Ce* value at the time of LOC was lower in ESKD patients; however, a significant difference was only detected between the Control and ESKD-CEB groups (Table 2). The statistical power to compare the difference was calculated 99.8% and 73.3% between Control group and ESKD group and between Control group and ESKD-CEB group, respectively. The pharmacodynamic model parameters and logistic regression curve depicting the relationship between the study groups and the probability of LOC are shown in Fig 2. The *Ce*_*50*_ values were estimated to be 4.56, 3.75, and 3.21 *μ*g/ml for the Control, ESKD, and ESKD-CEB groups, respectively.

**Table 2:** Effect-site concentration (*Ce*), total amount of propofol and bispectral index (BIS) at loss of consciousness.

	Control	ESKD	ESKD_CEB	*P*
*Ce (μ*g/ml)	4.0 (3.5–5.0)	4.0 (3.0–4.0)	3.0 (2.5–3.5)*	0.009
Total amount (mg)	124.0 (99.2–152.0)	94.5 (68.5–120.0)	81.0 (62.3–95.0)*	0.004
BIS	77.0 (68.0–81.0)	71.0 (65.0–77.0)	73.0 (68.0–84.0)	0.567

Values expressed in median (25th–75th percentiles). ESKD, end stage kidney disease; CEB, cervical epidural block.

*There is significant difference vs Control (*P* < 0.05).

## Discussion

In our small study, propofol Ce and Ce50 values were lower after infusion to achieve loss of consciousness than controls in ESKD patients who had received a CEB. In ESKD patients without a CEB, lower Ce50 was not statistically significant although likely was of clinical significance.

The incidence of ESKD requiring dialysis or kidney transplantation has increased with the increasing prevalence of hypertension and diabetes in the elderly population [11]. Accordingly, ESKD patients required to undergo surgical procedures are also increasing. Anesthesiologists should be aware of the anesthetic consideration of these patients. It is generally accepted that renal clearance decreases due to decreased estimated glomerular filtration rate in ESKD patients, which may affect the pharmacokinetics of the drugs cleared by the kidneys. However, many drugs, metabolized by the liver, are also known to affect drug distribution, metabolism and transport in ESKD patients and these pharmacokinetic alterations are thought to be due to the effects of uremia [12]. Changes in the biochemical function of a drug may be induced by uremic toxins and have various clinical manifestations [11, 13]. Dialysis is initiated in ESKD patients to treat uremic symptoms caused by toxic solutes; however, conventional dialysis does not eliminate the symptoms because it can only remove two-thirds of the total urea and a limited number of solutes [14].

Propofol, a drug that is not cleared by the kidneys, is primarily metabolized through O-glucuronidation in the conjugation pathway, or by oxidation via cytochrome P450 (CYP) in the liver, and is excreted in the urine [12, 15, 16]. This series of metabolic steps can be altered by uremic compounds that downregulate CYPs, or uremic toxins that act as competitive inhibitors interfering with CYP enzyme activity [4, 12]. In addition, the PTH level increases, and the inflammatory response is activated in ESKD patients [17]. Michaud et al. demonstrated that PTH and inflammatory cytokines are the main factors downregulating CYP in rats [5]. As a result, the propofol dose requirement in patients with ESKD can be difficult to predict and is expected to be different from that in patients with normal kidney function.

Among the serum proteins, propofol binds strongly to albumin and hemoglobin [16–18]. The protein binding of drugs changes in ESKD patients due to the effect of uremic substances, which act as protein binding inhibitors [12]. Moreover, anemia caused by a relative deficiency of erythropoietin is a common complication in ESKD patients [19]. The dosage of propofol should theoretically be reduced in patients with uremic symptoms and low levels of albumin or hemoglobin, as the fraction of free propofol increases.

Gasperi et al. [20] determined that the mean blood propofol concentration in ESKD patients was significantly lower than that in normal controls when inducing anesthesia with a bolus of 2 mg/kg propofol. Goyal et al. [9] concluded that the appropriate induction dose of propofol is higher in ESKD patients due to hyperdynamic circulation caused by anemia.

However, Ickx et al. [6] reported that the mean propofol blood concentration in ESKD patients did not differ significantly from that in a normal control group during slow induction by continuous infusion. Our results are similar to those of Ickx et al. In our study, Although the Ce was not different in ESKD patients, the Ce50 was lower by 0.81 *μ*g/ml, which although not statistically significant is likely a notable clinical effect. The reason for the lack of difference in the Ce of ESKD patients may be the slow infusion of propofol, which could have interfered with the hyperdynamic circulation.

There are several limitations in this study. First, although it has proper power for comparison, our study has small sample sizes. Second, weight, height, and BMI in ESKD patients were lower than those of patients with normal kidney function, and the ESKD-CEB group was older than the control group. The pharmacokinetics of propofol could be influenced by those. But we used TCI pump using the Schnider model. Schnider model include these parameters as covariates and improved the performance [10, 21]. Therefore, we presumed that the influence of the differences may be accounted.

Neuraxial anesthesia-induced sensory deafferentation reduces the demand for inhalation and intravenous anesthetics [22, 23]. Several studies have shown that epidural blockade reduces the propofol induction requirement [24, 25]. In this small study, we identified a decrease in the Ce and Ce50 values of ESKD patients who received CEB. However, there were no statistical differences between the two ESKD groups.

In conclusion, in our study, the Ce50 values were 3.75 and 3.21 μg/ml for the ESKD and ESKD-CEB groups, respectively. These values were lower 0.81 and 1.35 μg/ml compared with control group (4.56 μg/ml). Therefore, when inducing anesthesia in ESKD patients, we recommend using an initial dose similar to that of patients with normal kidney function, or rather starting with a lower dose.

## Supporting information

S1 Appendix Control file of the loss of consciousness pharmacodynamic model. (DOCX)
