# Supplementary material for: Potency of propofol for inducing loss of consciousness in end-stage kidney disease patients
Source: PLoS One. 2021 Aug 12;16(8):e0254520. doi: 10.1371/journal.pone.0254520 (PMC8360375; doi:10.1371/journal.pone.0254520)
Supplement: S1 Appendix — (DOCX) [file pone.0254520.s001.docx]

**Supporting information**

**S1 Appendix. Control file of the loss of consciousness pharmacodynamic model**

$PROBLEM 200

$DATA 20190702_Propofol_LOC.csv IGNORE=#

$INPUT ID TIME CE DV AMT ESRD CEB GRP BIS SBP DBP HR ASA AGE SEX HT WT LBM BMI ALB HB HCT PLT BUN CR EGFR NA K CL INR APTT

$PRED

TH1=THETA(1)

TH2=THETA(2)

TH3=THETA(3)

TH4=THETA(4)

IF(GRP.EQ.0) THEN

CE50=TH1*EXP(ETA(1))

CE50A=CE50

ENDIF

IF(GRP.EQ.1) THEN

CE50=TH2*EXP(ETA(2))

CE50B=CE50

ENDIF

IF(GRP.EQ.2) THEN

CE50=TH3*EXP(ETA(3))

CE50C=CE50

ENDIF

GAM=TH4*EXP(ETA(4))

PROB = CE**GAM/(CE50**GAM + CE**GAM)

IF(DV.EQ.1) THEN; LOC at DV=1, ROC at DV=0

IPRED = PROB; Probability of unconsicousness at DV=1

ELSE

IPRED = 1-PROB; Probability of consciousness at DV=0

ENDIF

W = 1

IRES = IPRED - DV

IWRES = IRES / W

Y = IPRED

$THETA ; #4

(0, 1) ; CE50_GRP0

(0, 1) ; CE50_GRP1

(0, 1) ; CE50_GRP2

(1, 3) ; Gamma

$OMEGA ; #3

0.2; IIV_CE50_GRP0

0.2; IIV_CE50_GRP1

0.2; IIV_CE50_GRP2

0 FIX ; IIV_GAM

$ESTIMATION NOTBT NOOBT NOSBT MAX=9999 SIGL=6 NSIG=2 LIKELIHOOD LAPLACE PRINT=5 NOABORT METHOD=1

$COVARIANCE PRINT=E

$TABLE ID ETA(1) ETA(2) ETA(4) ETA(4)

FILE=200.ETA NOPRINT FIRSTONLY NOAPPEND; PDx pop, fit4NM

$TABLE ID CE50A CE50B CE50C GAM

FILE=200.PAR NOPRINT ONEHEADER FIRSTONLY NOAPPEND; PDx pop

$TABLE ID CE IPRED IWRES

FILE=sdtab200 NOPRINT ONEHEADER; Xpose-delete SID and TAD for PsN, wfn

$TABLE ID CE50A CE50B CE50C GAM

FILE=patab200 NOPRINT ONEHEADER NOAPPEND; Xpose

$TABLE ID AMT AGE HT WT LBM BIS SBP DBP HR BMI ALB HB HCT PLT BUN CR EGFR NA K CL INR APTT

FILE=cotab200 NOPRINT ONEHEADER NOAPPEND; Xpose

$TABLE ID ESRD CEB GRP ASA SEX

FILE=catab200 NOPRINT ONEHEADER NOAPPEND; Xpose
